# Supplementary material for: Application of temperature-responsive HIS-tag fluorophores to differential scanning fluorimetry screening of small molecule libraries
Source: Front Pharmacol. 2022 Nov 24;13:1040039. doi: 10.3389/fphar.2022.1040039 (PMC9729254; doi:10.3389/fphar.2022.1040039)
Supplement: Supplementary file 1 [file DataSheet2.PDF]

Supplementary Material

Supplementary Figure 1 **(A)** nanoDSF backscattering readout for BbHtrA S/A in the presence of increasing concentrations of DMSO. Lines represent the means of three biological replicates with dotted lines showing the standard deviation. **(B)** nanoDSF thermograms for BbHtrA S/A in the presence of increasing concentrations of DMSO. Lines represent the means of three biological replicates with dotted lines showing the standard deviation. **(C)** HIS-DSF thermograms for BbHtrA S/A in the presence or absence of 50 mM EDTA and 250 mM imidazole. Lines represent the means of three biological replicates with dotted lines showing the standard deviation. **(D)** The Pearson r correlation coefficient shows significant correlation ($p < 0.005$) between the HIS-DSF EC_{50} and nanoDSF T_{onset} parameters for the confirmed hits.

Supplementary Table 1 Results of the HIS-DSF primary screen runs after analysis with Roche TSA software.
