# Supplementary figures and images for: Application of temperature-responsive HIS-tag fluorophores to differential scanning fluorimetry screening of small molecule libraries
Source: Front Pharmacol. 2022 Nov 24;13:1040039. doi: 10.3389/fphar.2022.1040039 (PMC9729254; doi:10.3389/fphar.2022.1040039)

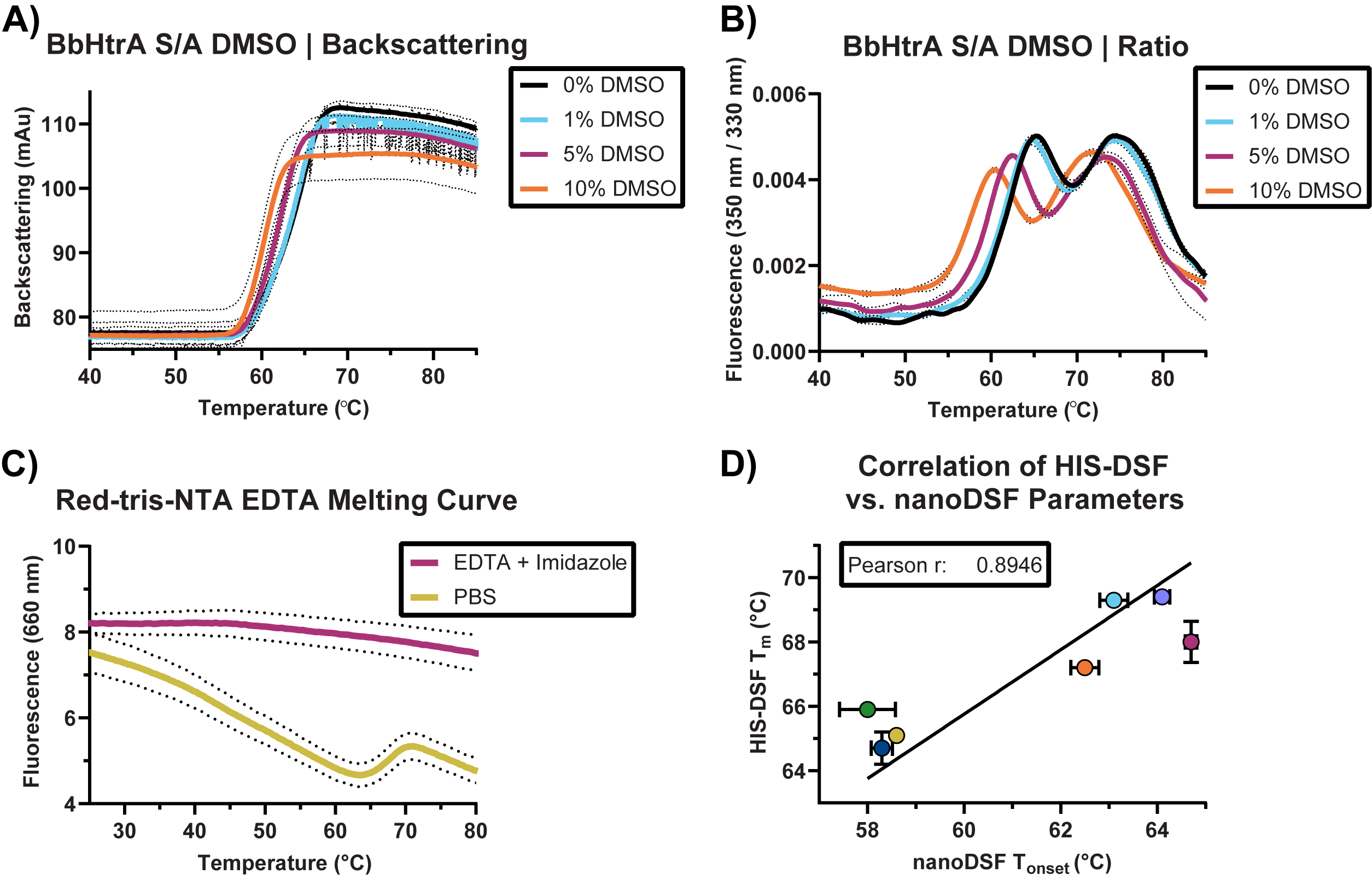

Supplement: Supplementary file 2 [file Image1.TIF]
